# Assessment of Etiology of Elephantiasis and Its Associated Risk Factors in Jeldu District, West Shoa, Ethiopia

**DOI:** 10.1155/2021/5551637

**Published:** 2021-04-15

**Authors:** Ararsa Negasa, Mebrate Dufera

**Affiliations:** ^1^Jeldu Preparatory School, West Shoa Zone, Gojo, Jeldu, Ethiopia; ^2^Department of Biology, College of Natural and Computational Sciences, Wollega University, Nekemte, Ethiopia

## Abstract

Elephantiasis is the main cause of tropical lymphoedema in Ethiopia. The aim of the current study was to assess the etiology of elephantiasis and its associated risk factors. Cross-sectional community-based study was carried out from March to May 2020 in seven purposively selected villages of Jeldu district. Identified suspected cases of elephantiasis in those villages were interviewed, clinically observed, and serologically examined using filariasis test strip kits at their respective houses during day time. A midnight blood sample was obtained from all FTS positive cases for microscopic examination of *Wuchereria bancrofti* larva, microfilariae. From a total of 105 identified elephantiasis cases, 69.5% were podoconiosis cases and 30.5% were LF cases. Among 32 serologically positive cases, only 15.6% cases were found positive by parasitological blood diagnosis. Considerable cases of podoconiosis (37%) were at age range 26–40 years, whereas smallest cases (16.4%) were at 10–25 years. Among cases of podoconiosis and LF, 77 subjects have been developed overt chronic leg(s) swelling; 85.7% of them showed bilateral swelling below knee and 14.3% of them showed unilateral swelling with stage II swelling 41.1%. Regarding risk factors, odds of podoconiosis was greatly lower in participants who washed their legs daily when compared to those who washed their legs sometimes (*P*=0.002). Odds of LF was higher in people who used bed nets/IRS and they were more protected than those who did not use bed nets/IRS (*P*=0.03). Odds of LF was high in cases farming besides rivers and/or perform irrigation work (*P*=0.003). The highest silicon concentration 4.65 mg/10 gm in Urgaha is coinciding with the highest podoconiosis cases (23.3%) of the village. Family history was a significant risk factor for the disease (*P* ≤ 0.001). Age (26–40 years), sex, and leg hygiene were also strong risk factors. Both etiologies of elephantiasis, LF and podoconiosis, are geographically overlapped in Jeldu district.

## 1. Introduction

Lymphatic filariasis (LF) is a neglected tropical disease that persists in developing countries and impoverished communities throughout Sub-Saharan Africa, Asia, South, and Central America and Pacific Island nations. Globally, it has been estimated that more than 1.3 billion people, most of whom are the world's poorest, are at risk for contracting LF, with 120 million infected and about 40 million disfigured or incapacitated people by the disease [1]; more than one-third of these are in Sub-Saharan Africa. Among Sub-Saharan Africans, Congo, Nigeria, Tanzania, Rwanda, Kenya, and Ethiopia are at risk of LF [2]. Elephantiasis represents a major public health problem in tropical and subtropical regions of the world, which is characterized by the thickening of the skin and underlying subcutaneous tissues, especially in the legs and male genitals and female breasts, causing permanent disability [3, 4]. Historically, in Ethiopia, the first case of LF was reported in 1971 [5]. Entomological investigations (human landing catch followed by dissection of mosquito) in Gambella show that *Anopheles gambiae* s.l. (probably *A. arabiensis*) and *A. funestus* were the main vectors of *Wuchereria bancrofti*, with no evidence for the involvement of the culicine mosquitoes despite the high biting rates [5]. Additionally, [6] identified the five LF endemic districts in Ethiopia namely, South-Ari, Benatsemay and Selemago, Teltele, Simada, and Tach-Gayint.

Non-filarial elephantiasis (podoconiosis) is a chronic inflammatory, geochemical skin disease caused by prolonged exposure to irritant red clay soils derived from volcanic rocks and causes bilateral asymmetrical swelling of the lower legs. It is the second most common cause of tropical lymphoedema after lymphatic filariasis and is characterized by prominent swelling of the lower extremities, which leads to disfigurement and disability [7]. Globally, the disease occurs in highland areas of tropical Africa, Central America, and northwest India and is related to poverty. It is estimated to affect 4 million people worldwide [8]. Podoconiosis which is common in areas of particular soil composition has been reported from Ethiopia, Uganda, Kenya, Tanzania, Rwanda, Burundi, and the Sudan where its distribution overlaps that of bancroftian filariasis [8, 9]. Furthermore, podoconiosis was reported from Gojjam, Northern Ethiopia by [10] during the markets survey in 1969; there were more than 100,000 cases in Ethiopia at the time.

Generally, recent assessments suggest that Ethiopia is projected to bear one-fourth (25%) of the global burden of podoconiosis, with up to 1 million cases of podoconiosis existing in Ethiopia [11]. LF also exposes an estimated 5.7 million people at risk of infection [12]. Therefore, these two neglected tropical diseases apparently lead to elephantiasis and therefore have fundamental public health importance [3]. Based on this information, this study is primarily designed for further investigation of cases, etiologies, and associated risk factors of elephantiasis in Jeldu district, West Shoa, Ethiopia.

## 2. Methods

### 2.1. Study Area

**Figure 1:**
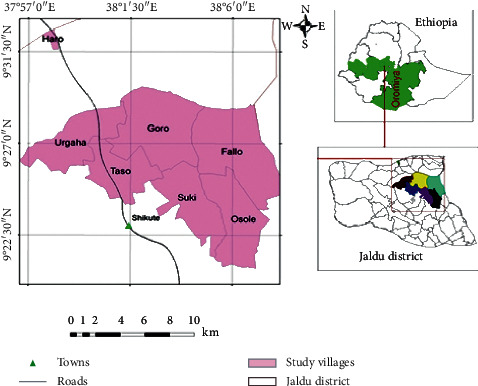
The study area map, Jeldu district, West Shoa, Ethiopia.

A cross-sectional community-based cases survey was conducted from March to May 2020 in seven purposively selected villages: Fallo, Goro, Haro, Osole, Suki, Taso and Urgaha of Jeldu district, West Shoa, Ethiopia. The district is located 114 km far from Addis Ababa and situated between 9°22′30″ to 9°31′30″ N latitudes and 37°57′0″ to 38°6′0″E longitudes direction (Figure 1).

### 2.2. Clinical, Serological, and Parasitological Examinations

Primary data collection was conducted in three phases. In the first phase, a pilot study was conducted on the study area one week prior to actual data collection to identify the presence of elephantiasis. In the second phase, uniquely triangulated actual data collections were maintained: elephantiasis clinical picture observation, responses from semi-structured questionnaire, and serological blood examination by FTS by collecting 75 *µ*L blood from ring finger of each participants. Suspected cases were also clinically observed for signs of the limb lymphoedema and then lymphoedema severity was graded and outlined by [13].

On the third phase, those cases who were found positive by FTS were re-sampled and the blood was taken from each participant from their ring finger at night between 8 p.m. and 4 a.m. at their respective houses. Nocturnal blood collection was carried out because of nocturnal periodicity of the parasite [14, 15]. About 40*μ*L finger prick blood was collected and stained to detect microfilariae. Microfilariae (no microfilariae/40 *μ*L) were also counted by two laboratory professionals. Types and physical and chemical properties of the soil are fundamental risk factors for occurrence of podoconiosis [16]. Therefore, randomly collected 300 gm of soil samples from each village was coded and transported to Holeta Agricultural Research Center (HARC) West Shoa, Ethiopia, for the mineral analysis.

## 3. Results

### 3.1. Sociodemographic Characteristics

Among a total of 392 study participants, 203 (51.8%) males and 189 (48.2%) females were interviewed as suspected cases of elephantiasis in the seven villages of the district, and 13 suspected individuals refused to give their blood sample. Therefore, clinical examination and serological blood diagnosis using FTS were done for 379 individuals, 194 (51.2%) males and 185 (48.8%) females, during second phase of the study. Of those participants, 55 (14.5%) suspected available cases were selected from Fallo Village while 36 (9.5%), 62 (16.4%), 60 (15.8%), 57 (15%), 60 (15.8%), and 49 (12.9%) were selected from Goro, Haro, Osole, Suki, Taso, and Urgaha, respectively. From a total of 105 identified elephantiasis cases, 73 (69.5%), 48 (45.7%) males and 25 (23.8%) females, were podoconiosis cases and 32 (30.5%), 12 (11.4%) males and 20 (19.1%) females, were LF cases. However, co-infection was not detected.

Considerable cases of podoconiosis (37%) were at age range 26–40 years, whereas smallest cases (16.4%) were at 10–25 years. Males (65.7%) were more affected than females (Table 1).

### 3.2. Physical (Clinical) Observation

The physical observation was made for all 379 individuals by two clinical nurses in response to identify the etiologies of elephantiasis on the basis of their clinical algorithm and grading the severity of swelling. Among those participants, 77 (19.5%) people were developed observable legs swelling and identified as elephantiasis cases by ruling out other encountered lymphoedema like leprosy, tungiasis, and other skin disorders. Of the 77 people, leg swelling of 64 (83.1%) podoconiosis cases and 2 (2.6%) LF cases were bilateral symmetry below knee. However, breast swelling and scrotal swelling (hydrocele) which are the best feature of LF were not observed. Furthermore, of 32 LF cases only 4 (5.2%) individuals developed observable leg swelling. Sites and type of swelling have strong statistical association with elephantiasis (*P* ≤ 0.001) (Table 2).

Of the two etiologies of elephantiasis identified from the study area, participants affected by podoconiosis developed overt chronic swelling while most LF positive cases seemed asymptomatic; only four individuals developed stage I swelling. Of four stages of lymphoedema identified among podoconiosis confirmed cases, stage IV was very rare (2.7%) but stage II was common 30 (41.1%). When severity stages of podoconiosis were considered relative to age groups, stages of podoconiosis showed significant association with age groups (*P*=0.02). The majority of the cases also suggested that they got reduction (decrease) in the size of their swelling after wearing covered shoes (Table 3).

**Figure 2:**
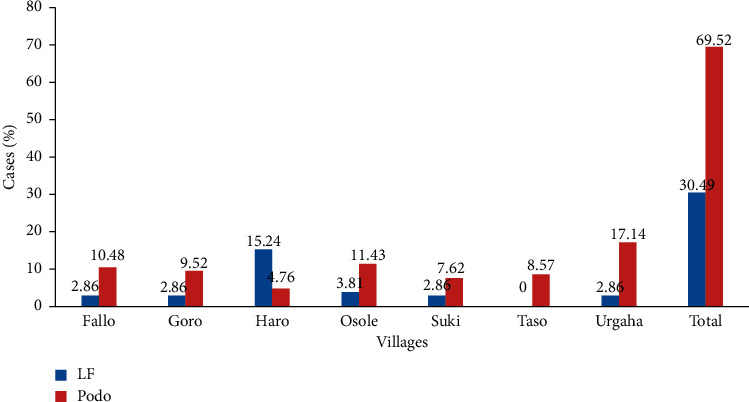
Distribution of LF and podoconiosis within the study villages (*n* = 105), Jeldu district, West Shoa, Ethiopia.

Regarding the distribution of LF and podoconiosis within the villages, most (17.14%) cases of podoconiosis were reported from Urgaha Village, while most (15.24%) cases of LF were from Haro Village, and least (4.76%) cases of podoconiosis were recorded in Haro Village. LF case was not recorded in Taso (Figure 2).

### 3.3. Serological and Parasitological Blood Examination

In the third phase of the study, cases that became positive by FTS were re-sampled for parasitological blood diagnosis, double-checked. Parasitological (thick blood smear) examination was performed for all FTS positive individuals during nighttime. Fortunately, of those 32 positive cases only 5 (15.6%) individuals, 3 (9.4%) females and 2 (6.2%) males, were identified positive for larva of *Wuchereria bancrofti* microfilariae (Table 4).

### 3.4. FTS Kits Intensity and Microfilariae Density

Although significance differences were not observed in both sex and age groups, higher mean of mf/40 *µ*L (5) was observed in females and with regard to age, mean of mf/40 *µ*L (4) was observed in age ranges 41–55. Intensity of the kits was also graded based on redness of the test lines; majority of cases 19 (61.3%) recorded low intensity (Table 5).

In addition to clinical, immunological, and parasitological findings, soil mineral analysis was carried out to diagnose podoconiosis; therefore, concentration of aluminium, iron, potassium silicon, and calcium was determined in seven soil samples taken from each selected village. The highest silicon concentration (4.65 mg/10 gm) in alkaline soil (pH = 7.5) of Urgaha is matched with the highest podoconiosis cases (17) (23.3%) of the village similarly; the highest iron concentration (8.71 mg/10 gm) in alkaline soil (pH = 7.4) of Osole coincided with higher podoconiosis cases (14) (19.2%) of the village; conversely, the least iron concentration (2.93 mg/10 gm) in acidic soil (pH = 6.7) of Haro was consistent with least podoconiosis cases (5) (6.8%) of the village (Table 6).

In the determination of the risk factors associated with podoconiosis, multivariable logistic regression analysis shows that family history (genetics) was a strong risk factor for the disease (AOR = 16.8,95% CI = 8.2–34.5, *P* ≤ 0.001). Age (26–40 years), sex (male), and leg hygiene were also strong risk factors (Table 7).

Among the determining factors identified with regard to LF, odds of LF were high in cases farming besides rivers and/or performing irrigation work (AOR = 3.3, 95% CI = 1.49–7.3, *P*=0.003). Bed net users also showed statistically significant association with LF (*P*=0.03) (Table 8).

## 4. Discussion

Apparently, the present study aimed to investigate the etiology of elephantiasis and associated risk factors of the disease using structured questionnaire, clinical, serological, and parasitological blood examination and soil analysis. In the present study, a screening made by clinical examination following checking of suspected cases using FTS kits identified that the majority of the people developed overt chronic leg(s) swelling with bilaterally asymmetric; the ratio of unilateral to bilateral is 1 : 6. This is consistent with previous study conducted by [17] who reported 1 : 8 from integrated cases of LF and podoconiosis in 20 co-endemic districts of Ethiopia and 1 : 16 from Midakegn district, Central Ethiopia [18]. The present study identified that none of elephantiasis cases shows any symptom of hydrocele which is the main clinical manifestation of LF. In contrast, [19] had reported 20.7% hydrocele and 0% lymphedema in southwestern Ethiopia, Gambella region, whereas 34% hydrocoele and 8.5% lymphedema were reported from Kenya [20]. The difference might be due to the difference in the favorability of the environment for mosquito breeding.

Multivariable logistic regression analysis shows that sex has significant statistical association with podoconiosis in agreement with a nationwide study in Ethiopia [21] and a case control study in Northern Ethiopia shows that females were associated with increased risk of podoconiosis [22]. The present study identified that cases of podoconiosis were more in males than in females with male-to-female ratio of 1.9 : 1 which was in contrast to [23] in Wayu Tuka district, eastern Wollega, in which male-to-female ratio was 1 : 1.58 and 0.98 : 1 in Gojjam, Northern Ethiopia [22]. The risk difference could be due to occupationally (agricultural) linked trauma to the feet (poor habit of wearing shoes) and the cumulative effect of long-term exposure to the soil of the area during farming [24, 25]. Present finding also unfolds that more than half cases of podoconiosis were found in age range 26–40 years similarly; 78% of cases in Midakegn district, Central Ethiopia, were found in age range 21–60 years [18]. In the present study, of four stages of podoconiosis with characteristics of itchy swelling, shallow skin folds, and mossy lesion were identified by clinical nurses; stage II was the most common, followed by stage I, stage III, and stage IV, respectively. This agreed with nationwide study in Ethiopia [21, 25] from Dano district, Central Ethiopia. The present study also shows that stage IV was absent in lower age and appeared at higher age (56–70) accompanied with secondary infection; furthermore, age groups and stages of lymphoedema have significant statistical association and agreed with an idea that incidence and severity of podoconiosis increase with age, likely due to cumulative exposure to irritant soil [24, 26].

The current study also suggested that majority of cases of podoconiosis had developed decrease in the size of swelling after wearing covered shoes compared to those who did not wearing shoes; wearing shoes and reduction of swelling have significant statistical association. In line with empirical evidence from Southern Ethiopia, it shows a reduction in leg circumference and an improvement in clinical stage following the use of a simple lymphoedema management method including regular use of footwear, washing of feet with soap, water and an antiseptic, and compression bandage or elevation of legs at night [27]. Multivariable logistic regression analysis also shows that family history and leg hygiene were significantly associated with podoconiosis. In line with [28], odds of podoconiosis were 16.8 times higher on family history with the disease than those who are not. This might be due to the effect of genes in the development of podoconiosis; this result still requires further genetic analysis. The risk of acquiring podoconiosis among participants who washed their leg daily was greatly lower compared to those who washed their leg sometimes. This might be due to the reason that keeping foot hygiene will detach the possible provoking agent away from susceptible hosts and it is consistent with the reports of [29].

In line with [16, 30], the present study indicates that highest cases of podoconiosis were identified from village with alkaline soil containing highest concentration of iron, aluminium, and silicon compared to those in village containing lower concentrations of those minerals. Therefore, iron, aluminium, silicon, and probably potassium are the predictors of podoconiosis in Jeldu district.

The present study shows positive cases by FTS kits were higher than those identified by microscope blood smear. The difference might be due to low sensitivity of microscope [31]. This study also indicates that majority of LF cases were females those with high intensity by FTS with female-to-male ratio of 1.7 : 1. The sex difference could be due to high metabolic burdens on females like pregnancy, lactation, and menstruation which probably decrease their immune status [32]. In the current work, multivariable logistic regression analysis for LF indicates that age, bed net, and irrigation farming have strong association with an occurrence of LF which is in agreement with a report from Congo [33].

## 5. Conclusions

The positive cases (30.5%) identified by both serological and parasitological blood diagnosis indicate the presence of lymphatic filariasis. Also, soil analysis and clinical examination of leg(s) swelling indicate (69.5%) the presence of podoconiosis in the study area. Based on this evidence, we conclude that both etiologies of elephantiasis, filarial elephantiasis and endemic non-filarial elephantiasis (podoconiosis), are available in Jeldu district. We recommend that integrated interventions and rapid re-assessment of these diseases should be taken into account to avail medications and treatment materials for the community.

## Figures and Tables

**Table 1:** Sex and age distribution of podoconiosis cases (*n* = 73) in Jeldu district, West Shoa, Ethiopia.

		Age groups (year)				Total (*n* = 73) *n* (%)
		10–25 (*n* = 12) *n* (%)	26–40 (*n* = 27) *n* (%)	41–55 (*n* = 21) *n* (%)	56–70 (*n* = 13) *n* (%)	
Sex	Male	7 (9.6)	16 (21.9)	15 (20.5)	10 (13.7)	48 (65.7)
	Female	5 (6.8)	11 (15.1)	6 (8.2)	3 (4.1)	25 (34.3)

Leg	Single	1 (1.37)	3 (4.1)	2 (2.7)	3 (4.1)	9 (12.3)
	Both	11 (15.1	24 (32.9)	19 (26)	10 (13.7)	64 (87.7

**Table 2:** Type and sites swelling of elephantiasis cases (*n* = 77) in Jeldu district, West Shoa, Ethiopia.

	Elephantiasis			*P* value
	Podo (*n* = 73) *n* (%)	LF (*n* = 4) *n* (%)	Total (*n* = 77) *n* (%)	
*Sex*				
Male	48 (62.3)	1 (1.3)	49 (63.6)	
Female	25 (32.5)	3 (3.9)	28 (36.4)	0.01

*Site of swelling*				
Bilateral below knee	64 (83.1)	2 (2.6)	66 (85.7)	≤0.001
Unilateral below knee	8 (10.4)	2 (2.6)	10 (13)	
Unilateral above knee	1 (1.3)	0 (0)	1 (1.3)	

*Type of swelling*				
Fibrotic (hard)	67 (87)	4 (5.2)	71 (92.2)	≤0.001
Watery (soft)	6 (7.8)	0 (0)	6 (7.8)	

**Table 3:** Stages of podoconiosis cases (*n* = 73) in Jeldu district, West Shoa, Ethiopia.

Podo cases		Stages				Total (*n* = 73) *n* (%)	*P* value
		I (*n* = 25) *n* (%)	II (*n* = 30) *n* (%)	III (*n* = 16) *n* (%)	IV (*n* = 2) *n* (%)		
Sex	Male	15 (20.5)	19 (26.1)	12 (16.4)	2 (2.7)	48 (65.7)	0.04
	Female	10 (13.7)	11 (15.0)	4 (5.5)	0 (0)	25 (34.3)	

Age	10–25	6 (8.2)	4 (5.5)	2 (2.7)	0 (0)	12 (16.4)	
	26–40	5 (6.8)	14 (32.6)	8 (11)	0 (0)	27 (37)	0.02
	41–55	12 (16.4)	7 (9.6)	2 (2.7)	0 (0)	21 (28.8)	
	56–70	2 (2.7)	5 (6.8)	4 (5.5)	2 (2.7)	13 (17.8)	

**Table 4:** LF cases and the diagnostic tools in Jeldu district, West Shoa, Ethiopia.

LF cases		Diagnostic tools		Both (*n* = 5) *n* (%)
		FTS +ve (*n* = 32) *n* (%)	Microscope +ve (*n* = 5) *n* (%)
Sex	Male	12 (37.5)	2 (6.2)	2 (6.2)
	Female	20 (62.5)	3 (9.3)	3 (9.4)

**Table 5:** Intensity of FTS kits of LF cases (*n* = 32) with relative age and sex in Jeldu district, West Shoa, Ethiopia.

		Intensity of kits					
		Low (*n* = 19) *n* (%)	Medium (*n* = 7) *n* (%)	High (*n* = 6) *n* (%)	Total (*n* = 32) *n* (%)	Mean mf/40 *µ*L	*P* value
Sex	Male	8 (25)	3 (9.4)	1 (3.1)	12 (37.5)	3	0.27
	Female	11 (15.1)	4 (12.5)	5 (15.6)	20 (62.5)	5	

**Table 6:** Soil mineral types of the study villages in Jeldu district, West Shoa, Ethiopia.

Study villages	Type of minerals (mg/10 gm)					pH	Podoconiosis cases *n* (%)
	Aluminium (Al)	Iron (Fe)	Silicon (Si)	Potassium (K)	Calcium (Ca)		
Fallo	0.98	4.05	2.87	5.38	4.55	7.3	12 (16.4)
Goro	0.83	3.96	3.51	9.44	7.21	7.1	7 (9.5)
Haro	0.55	2.93	0.72	8.23	10.41	6.7	5 (6.8)
Osole	1.67	8.71	3.42	7.12	5.12	7.4	14 (19.2)
Suki	1.07	3.28	1.22	12	3.8	6.9	8 (11)
Taso	0.82	3.82	1.36	6.43	2.33	7.1	10 (13.7)
Urgaha	2.21	4.64	4.65	1.39	10.5	7.5	17 (23.3)

**Table 7:** Multivariate logistic regression analysis of associated risk factors of podoconiosis cases (*n* = 73) in Jeldu district, West Shoa, Ethiopia.

Factors		Cases *n* (%)	COR (95% CI)	AOR (95% CI)	*P* value
Sex	Male	48 (65.7)	0.45 (0.24–86)	2.2 (1.16–4.19)	0.02
	Female	25 (34.3)	1	1	1

Age	10–25	12 (16.4)	0.72 (0.24–2.1)	1.39 (0.46–4.18)	0.56
	26–40	27 (37)	0.37 (0.15–0.9)	2.7 (1.1–6.8)	0.03
	41–55	21 (28.8)	0.60 (0.24–1.5)	1.67 (0.66–41)	0.28
	56–70	13 (17.8)	1	1	1

Occupation	Farmers	65 (89)	0.51 (0.17–1.6)	1.95 (0.6–6.0)	0.24
	Students	2 (2.7)	1.94 (0.26–14)	0.51 (0.07–3.9)	0.52
	Others	6 (6.8)	1	1	1

Family history	Yes	51 (69.8)	0.06 (0.03–0.12)	16.8 (8.2–34.5)	≤0.001
	No	22 (30.2)	1	1	1

Leg hygiene	Yes	47 (64.4)	3 (1.5–60)	0.33 (0.17-0-6)	0.002
	No	26 (35.6)	1	1	1

**Table 8:** Multivariate logistic regression analysis of associated risk factors of LF (*n* = 32) in Jeldu district, West Shoa, Ethiopia.

Factors		FTS +ve *n* (%)	COR (95% CI)	AOR (95% CI)	*P* value
Sex	Male	12 (37.5)	1.5 (0.68–3.4)	0.66 (0.3–1.46)	0.30
	Female	20 (62.5)	1	1	1

Age	10–25	7 (21.9)	0.92 (0.24–3.7)	1.08 (0.27–4.3)	0.92
	26–40	6 (18.8)	1.1 (0.3–3.7)	0.94 (0.27–3.342)	0.90
	41–55	14 (43.8)	0.5 (0.17–1.5)	2.00 (0.66–6.0)	0.21
	56–70	5 (15.6)	1	1	1

Occupation	Farmers	27 (84.4)	0.46 (0.058–3.6)	2.2 (0.27–17)	0.46
	Students	4 (12.5)	0.27 (0.024–2.97)	3.75 (0.33–41)	0.28
	Others	1 (3.1)	1	1	1

Bed net	Yes	8 (25)	2.5 (1.1–6)	0.39(0.16–0.93)	0.03
	No	24 (75)	1	1	1

Drugs (DEC/IVM)	Yes	2 (6.3)	1	1	1
	No	30 (92.7)	1.24*E* + 10	—	—

Irrigation	Yes	19 (59.4)	0.3 (0.14–0.67)	3.3(1.49–7.3)	0.003
	No	13 (40.6)	1	1	1

## Data Availability

The dataset generated from patients' clinical record is not publicly available to protect patient confidentiality. Unidentifiable data can be obtained from the corresponding author upon reasonable request.
